# Continuing with “*…a heavy heart*” - consequences of maternal death in rural Kenya

**DOI:** 10.1186/1742-4755-12-S1-S2

**Published:** 2015-05-06

**Authors:** Rohini Prabha Pande, Sheila Ogwang, Robinson Karuga, Radha Rajan, Aslihan Kes, Frank O Odhiambo, Kayla Laserson, Kathleen Schaffer

**Affiliations:** 1 Independent Consultant, 7118 Willow Avenue, Takoma Park MD 20912, USA; 2 Kenya Medical Research Institute (KEMRI), Kisumu, Kenya; 3 LVCT Health, Tivoli Centre, P.O Box: 3294-40100, Kisumu, Kenya; 4 Johns Hopkins University Bloomberg School of Public Health, 615 N. Wolfe Street, Baltimore, MD 21231, USA; 5 International Center for Research on Women (ICRW), 1120 20th St NW, Suite 500 North, Washington, D.C. 20036, USA; 6 Global Disease Detection Program, Centers for Disease Control (CDC), 1600 Clifton Rd., Atlanta, GA 30329-4027, USA; 7 Family Care International, 588 Broadway #503, New York, NY 10012, USA

**Keywords:** maternal death, Kenya, infant survival

## Abstract

**Background:**

This study analyzes the consequences of maternal death to households in Western Kenya, specifically, neonatal and infant survival, childcare and schooling, disruption of daily household activities, the emotional burden on household members, and coping mechanisms.

**Methods:**

The study is a combination of qualitative analysis with matched and unmatched quantitative analysis using surveillance and survey data. Between September 2011 and March 2013 all households in the study area with a maternal death were surveyed. Data were collected on the demographic characteristics of the deceased woman; household socio-economic status; a history of the pregnancy that led to the death; schooling experiences of surviving school-age children; and disruption to household functioning due to the maternal death. These data were supplemented by in-depth and focus group discussions. Quantitative data on neonatal and infant survival from a demographic surveillance system in the study area were also used. Descriptive and bivariate analyses were conducted with the quantitative data, and qualitative data were analyzed through text analysis using NVivo.

**Results:**

More than three-quarters of deceased women performed most household tasks when healthy. After the maternal death, the responsibility for these tasks fell primarily on the deceased’s husbands, mothers, and mothers-in-law. Two-thirds of the individuals from households that suffered a maternal death had to shift into another household. Most children had to move away, mostly to their grandmother’s home. About 37% of live births to women who died of maternal causes survived till age 1 year, compared to 65% of live births to a matched sample of women who died of non-maternal causes and 93% of live births to surviving women. Older, surviving children missed school or did not have enough time for schoolwork, because of increased housework or because the loss of household income due to the maternal death meant school fees could not be paid. Respondents expressed grief, frustration, anger and a sense of loss. Generous family and community support during the funeral and mourning periods was followed by little support thereafter.

**Conclusion:**

The detrimental consequences of a maternal death ripple out from the woman’s spouse and children to the entire household, and across generations.

## Background

In 2013, there were an estimated 289,000 maternal deaths worldwide, down 45% from 1990 [1]. Despite this improvement, maternal mortality continues to be of concern, particularly in sub-Saharan Africa, which has the highest maternal mortality ratio of all world regions [1]. Yet, while there is a large body of research on *causes* of maternal mortality, research on its *consequences* for surviving household members, including children, is limited. This paper contributes to filling this gap by analyzing the consequences of maternal death for child health, child survival and household functioning in a region of Kenya.

The death of an adult family member can have significant negative impacts on children’s survival, health and schooling [2,3]. The death of a mother is particularly harmful [4-8], perhaps even more so when a mother dies during childbirth or postpartum. A randomized community trial in Nepal [9] found that maternal mortality was statistically significantly associated with early infant mortality, even after controlling for other relevant predictors. A qualitative study in Tanzania [10] described the impact of a maternal death on a range of survival, nutritional, health and other intergenerational outcomes for children, while research in rural Haiti found that a family with a maternal death had a 55% higher odds of experiencing a death to a child under age 12 years, but that there were no increased odds for child mortality in the case of non-maternal adult deaths. The authors of the Haiti study concluded “…a maternal death has a more significant impact on children and families than the non-maternal death of a woman of reproductive age” ([11]; p400).

Few studies analyze the impact of a maternal death on households as a whole. A recent exception in Tanzania showed that a maternal death had notable negative effects on family functioning in multiple ways: fathers were unable to sufficiently care for children, siblings were separated and sent to relatives for care, and the household suffered because of the loss of the “…crucial unremunerated care-giving and other household work that women perform which is essential to a functioning household” ([10]; pg. e71674). Such disruptions may ease once households adjust to their new situation [12], but longer-term studies to confirm this pattern are missing.

This paper extends the limited prior work by examining the consequences of maternal death for surviving household members, including children, in an impoverished rural part of western Kenya. A methodological innovation of this paper is the insertion of a retrospective, narrative approach into a quantitative survey to map disruptions in household functioning. Findings from this study are expected to contribute to the mounting evidence on the urgency to address maternal mortality.

## Methods

### Ethical approval

The study is a collaborative effort between Family Care International (FCI), the International Center for Research on Women (ICRW), and the KEMRI/CDC Research and Public Health Collaboration, a partnership between the Kenya Medical Research Institute (KEMRI) and the U.S. Centers for Disease Control and Prevention (CDC). The institutional review boards of ICRW, CDC and KEMRI approved the research protocols and consent processes.

### Study setting

**Figure 1:**
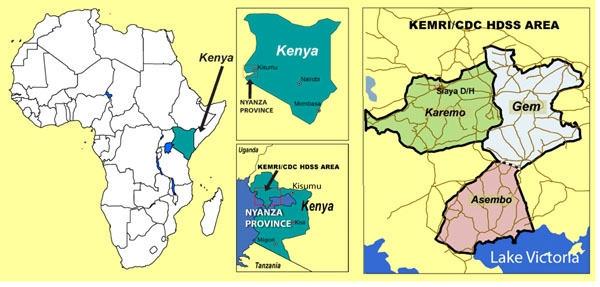
Map of Kenya, including study areas

This study was undertaken between 2011-2013 in Kenya. Recent estimates from the WHO show that the MMR in Kenya declined minimally between 1990 and 2013, from 490 per 100,000 births to 400, and that Kenya is one of 10 countries that together contributed 58% of global maternal deaths reported in 2013 [1]. Within Kenya, our study was conducted in Rarieda, Gem and Siaya sub-counties of Siaya County, which falls within the geographic region covered by KEMRI/CDC’s Health and Demographic Surveillance System (HDSS), established in 2001 (Figure 1).

This area has high levels of poverty, some of the highest HIV, tuberculosis and malaria rates in Kenya, some of the worst indicators for child and overall health status, and has been one of the most poorly served counties in terms of public health facilities [13,14]. Finally, maternal mortality is higher than in Kenya as a whole, at 740 per 100,000 live births between 2003-08 [15].

The study area is culturally homogenous, and over 95% of the population belongs to the Luo ethnic community. Society is polygamous. Families mostly live in separate homes arranged within a common homestead (*dala*), and up to three generations may cohabit the same homestead [16]. Women typically move to their husband’s homestead upon marriage.

### Research questions

In this paper we used a multi-method approach to examine two questions:

1. How do neonatal and infant survival vary by mother’s survival status?

2. How do household dynamics, functioning and welfare change after a maternal death and how do surviving adult household members cope?

### Research design

Our analysis uses surveillance, survey and qualitative data. Surveillance data from the KEMRI/CDC HDSS were used to identify households with a maternal death and births to women who died of maternal causes. The HDSS uses the WHO Verbal Autopsy method to identify maternal deaths, defined as deaths to women while giving birth or within 42 days after birth. We use this definition here when we refer to ‘maternal deaths’ or deaths from ‘maternal causes.’ We include women aged 15-49 years.

Each homestead in the HDSS area has been mapped using Global Positioning System (GPS) coordinates and each individual has been given a unique identification number. Information is collected on all births, deaths, causes of death (through verbal autopsy), pregnancy, pregnancy outcomes, morbidity, migration, education, and socioeconomic status. The verbal autopsy interviews are conducted by a team of trained community interviewers, with additional staff responsible for quality control.

This paper used two sets of data. To assess neonatal and infant mortality by maternal survival, we used a combination of HDSS verbal autopsy and data on births^a^ between 2003-11. We identified for our analysis 83 children born within 9 months of a maternal death who were still living when the maternal death occurred. We compared survival status till age 1 year of these births to two sets of matched controls: children born within 9 months of a death to mothers who died of non-maternal causes (57 live births), and live births to mothers who were still alive at the time of the survey (83 live births, randomly selected from the larger universe of live births to surviving mothers). Both sets of controls were matched to cases by timing of birth (women with children born within 2 months of the birth to the woman who died of maternal causes).

To assess household disruption, we conducted a quantitative survey between September 2011 and March 2013 to collect data on household socio-economic and demographic status. All households in the study area with a maternal death identified by the HDSS were surveyed. To minimize recall issues, we recruited households on a rolling basis as maternal deaths were identified. Out of respect for the trauma of a maternal death, we started enrolling households after a period of at least two months – but as soon as possible after that and up to 6 months– following the maternal death. We identified a total of 67 eligible households, of which 59 were surveyed. The remainder either refused or no appropriate respondent was found. Quantitative analyses presented from this survey are descriptive and do not include a control group.

A key methodological innovation of this study was the use of a retrospective, narrative design embedded within the quantitative survey to capture household disruption due to the maternal death. We here define ‘disruption’ as changes in allocation of different tasks among members of the deceased woman’s household before and after the maternal death. We started by asking which of a series of household, farming and other tasks the deceased woman did when she was still healthy. We then asked who else in the household had worked on each of these tasks with her. Next we moved to the situation after the death. We returned to each task for which the deceased woman was recorded as having been responsible, and asked who does this task now that the woman is deceased. We thus built a “disruption chain” illustrating the changes in the division of tasks among household members from before the maternal death to the time of the interview. Since we were unable to provide diaries or other methods to capture tasks in real time, we attempted to minimize recall errors by questioning the adult most likely to be knowledgeable about each type of household task in which we were interested. Our questionnaire was, therefore, constructed to allow different respondents for questions on different household tasks.

Finally, we conducted qualitative group discussions and in-depth interviews with a sub-set of surveyed households that had a maternal death, focusing on household disruption, childcare, emotional consequences and support mechanisms. In each participating household, the discussion occurred after the survey and within 6 months following the maternal death. Since the aim of the study was to capture the experiences and disruptions caused by a maternal death in the lives of different household members, and since households in our study area cohabit in homesteads, each discussion group comprised available and consenting surviving adults from a single homestead. The selection of households for the group discussions was largely determined by the availability of responding adults in the affected homestead. Where surviving adults comprised a single individual – typically the husband – we used the same guide to conduct an in-depth interview. Discussion moderators were trained by the study authors, and were instructed to refer participants, as needed, to KEMRI/CDC staff trained in counseling.

**Figure 2:**
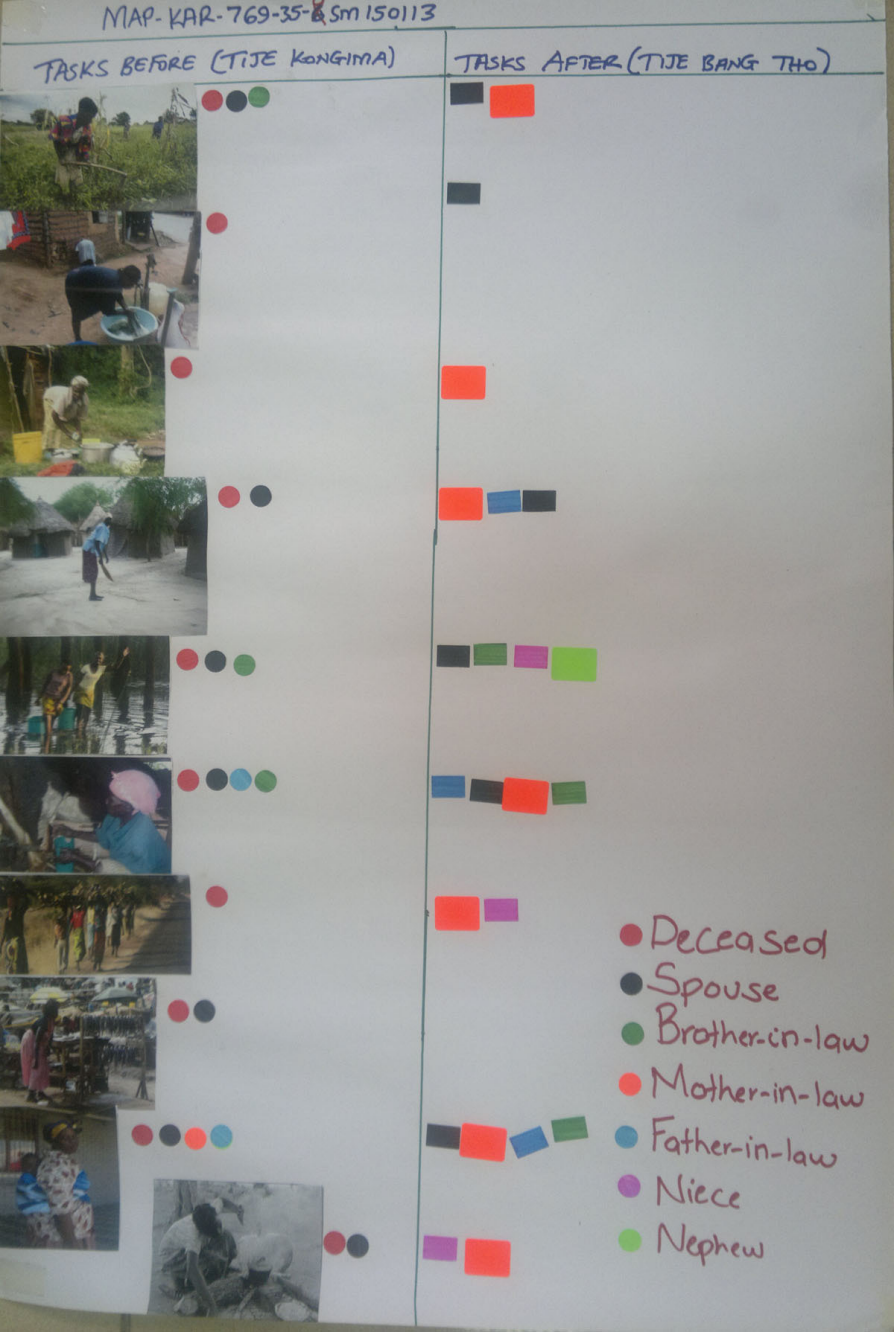
Illustrative pictorial representation of tasks and responsibilities (Task Mapping)

Following this approach, we conducted 8 group discussions with between 3 and 7 household members each and 3 in-depth interviews with husbands of women who died due to maternal causes. At each discussion, the research team shared with the participant(s) a range of pictures representing the day-to-day tasks in which women and households in this part of rural Kenya typically engage, such as farming, cooking, childcare, laundry, cleaning dishes, going to market, etc. Participants selected those pictures that accurately depicted the tasks in their household. Discussion moderators then engaged participants in a discussion on the main tasks the deceased woman did when she was alive and healthy, who helped, and who mainly does the task after the maternal death. Each person in the household was represented on a resulting ‘task mapping’ chart with a different color. Participation in tasks before the maternal death was represented by a circle, and participation after the maternal death was represented by a square. Once the chart was completed, moderators invited discussion participants to talk about what they saw on the chart, thus generating a discussion on division of tasks, changes after the maternal death, and participants’ emotions about the maternal death. Figure 2 presents an example of a completed task map.

Qualitative data transcripts were audio-recorded, transcribed into Luo by the interviewers and translated by KEMRI/CDC staff into English. Translated transcripts were analyzed using NVivo Version 10. The research team generated a series of hierarchical codes based on the issues of interest. Two of the researchers separately coded two transcripts each based on these codes. The inter-coder reliability was over 90%. Quantitative data were analyzed using Stata 11.0 and 13.0 and SAS Version 9.2.

## Results

### Background characteristics

The median age of surveyed women who died from maternal causes was 26 years (Table 1). About two-thirds of women were in monogamous marriages/cohabitation, about 24% were not married and the rest were in polygamous marriages/cohabitation. A little more than one quarter of deceased women had secondary schooling or higher, while three quarters or more had at least primary schooling, and only a small percentage reported no schooling at all. More than half the deceased women had been farmers in the 12 months prior to the survey. Somewhat less than half (43.5%) had been self-employed (mainly running a small store or market stall) and a minority had worked for wages.

**Table 1:** Background characteristics of surveyed women who died of maternal causes (2011-13)

Characteristic	Percent
***Median age (years)***	26.0

***Current marital status***	

Not married	23.7

Monogamous (married or cohabiting)	61.0

Polygamous (married or cohabiting)	15.3

***Highest education level***	

None/don’t know	1.7

Up to primary complete	69.5

Secondary or higher	28.8

***Economic activity in last 12 months***	

Wage or salary work	8.7

Farming	56.5

Own business/self-employment	43.5

***Household characteristics (N=123)***	

Household size (average members per household)	5.6

Percent of household members who are children 0-5 years	27.4

Percent of household members who are school-age (5-24 yrs)	52.2

**Total maternal deaths included in analysis**	59

### Implications of a maternal death for the household

Maternal death is the third most common cause of death for adult women in our study area, following HIV/AIDS and pulmonary tuberculosis^c^. However, unlike deaths from HIV/AIDS or tuberculosis, maternal deaths are likely to be unforeseen, and thus households are unlikely to have made financial or other arrangements to deal with the resulting disruption. When the maternal death leaves in its wake a newborn, the household may be left scrambling even more. For these reasons, despite the fact that maternal death is not the leading cause of death, it is likely a critical one in terms of its immediate impact on surviving household children and adults. Below we describe some of these impacts.

#### Household dissolution

**Figure 3:**
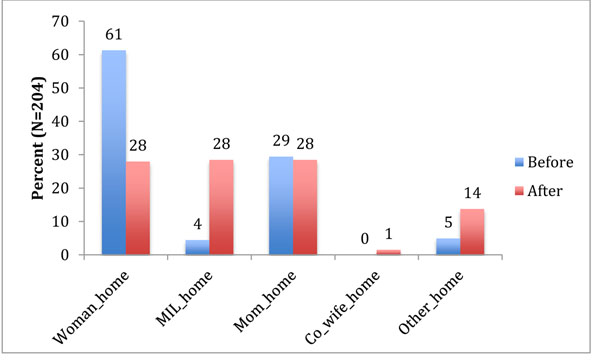
Where household members ate before and after the maternal death

Maternal deaths often triggered household dissolution, defined by where household members ate. Before the maternal death, the majority of household members (61.3%) ate in the woman’s own household. After she died, only 28% continued eating in her household. Mothers-in-law of the deceased women saw the largest intake of additional family members to feed: the proportion of family members that ate at a mother-in-law’s house jumped from under 5% of sample household members before the maternal death to more than a quarter (28%) after the maternal death (Figure 3).

#### Disruption in household and farming tasks

Across the developing world, women are largely responsible for the family and home. Our study area was no different, and women did most household tasks, sometimes in their natal family’s home as well as their own household. A majority of survey respondents (close to 80%) reported that the deceased woman cleaned the homestead, did laundry, cooked, and fetched water. Three-quarters or more reported she went to the market to buy daily supplies, and took care of children.

**Figure 4:**
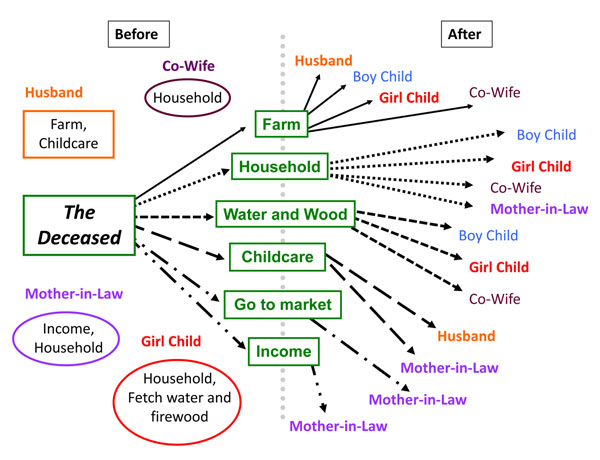
Illustration of disruption in one sample household

It is not surprising, therefore, that a maternal death created significant disruption in household functioning. Surviving adults in the household typically had to cover the gamut of activities that the deceased woman used to perform. The household illustrated in figure 4 exemplifies the nature of this change in household responsibilities. The patterns in figure 4 show both, how women were responsible for most tasks as well as the fact that it took many people to do the tasks the woman used to do almost single-handedly before her death.

**Figure 5:**
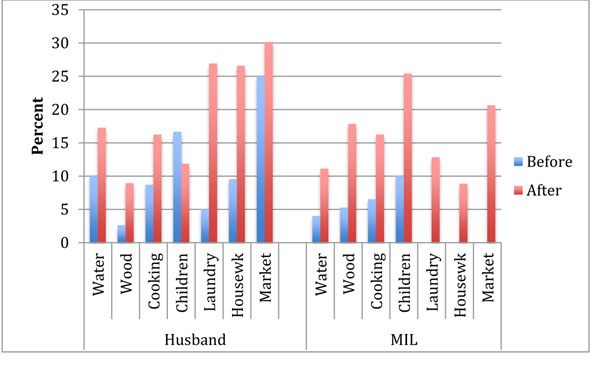
Participation of husbands and mothers-in-law in household tasks before and after the maternal death

Husbands, in particular, took on extra, unfamiliar responsibilities, such as cooking, laundry and cleaning the house (Figure 5). As one husband noted:

“…[I] am used to going to the farm early, but when she was gone it was a must that I make sure that those children have had something to drink…When I come back from the farm, I need to wash the clothes, I need to wash them [children], I also need to find them food; all this on how many people? Me, just one person…[with the grandmother’s help].”

The resulting fatigue was often debilitating, prompting one husband to say that by the end of the day, “*paro to nabolo chien*” (he was so tired that he forgot to have thoughts).

The increase in the range of responsibilities was perhaps even heavier for mothers-in-law of the deceased. Before the maternal death, mothers-in-law were largely not involved in their daughters-in-law’s work of cleaning house, doing laundry, or going to the market. After the maternal death, in several families mothers-in-law picked up these tasks (Figure 5).

Though such care was recognized as a part of family responsibilities, it was clearly a strain on the elderly women. For instance, twice as many mothers-in-law as earlier were involved in childcare, even as their other household work increased because of the maternal death. As one mother-in-law pointed out, she was *“…forced to do things quickly so that she [deceased’s child] does not sleep before she eats. If I don’t cook early then that day she [deceased’s child] sleeps hungry*...” Mothers of the diseased woman were similarly affected, noting that they again had been *“given the burden of a young last born…”* even though it had been a while since their own children had grown up.

#### Ripple effects of maternal death: the “disruption chain”

The shifts in household responsibilities illustrated by figures 4 and 5 and described above created a “disruption chain” whereby household members’ pre-existing tasks were affected as they took on the deceased woman’s responsibilities. As a result, these pre-existing tasks suffered, such as wage labor or agricultural activities. Work was often disrupted because of the now limited time available. As one mother-in-law noted:

“When this girl [the deceased woman] was not there, and I have to come back [from the farm] and make for them tea, or I even make for them porridge so that they can take, that is when I go back to the farm. When it reaches one o clock, I come back and see that they have some ‘ugali’ [a staple food made of maize flour cooked in boiling water until stiff] and vegetables, I leave when they have already eaten, then I go back to the farm.”

This situation negatively affected household income. Still others were pushed further into poverty as they had to pay casual labor to work on their fields; in some cases, even with casual labor families were forced to leave some land fallow, as this husband pointed out:

“Yes, like the land that we were cultivating up there, right now it is lying fallow because I cannot manage working on the two of them even the one down there. So I am forced to cultivate the little that I know that I can weed, I can manage to do all the work on that farm. I am forced to get some casual laborers who then plant for me….”

### Implications of a maternal death for orphaned children

#### Neonatal and infant mortality

**Figure 6:**
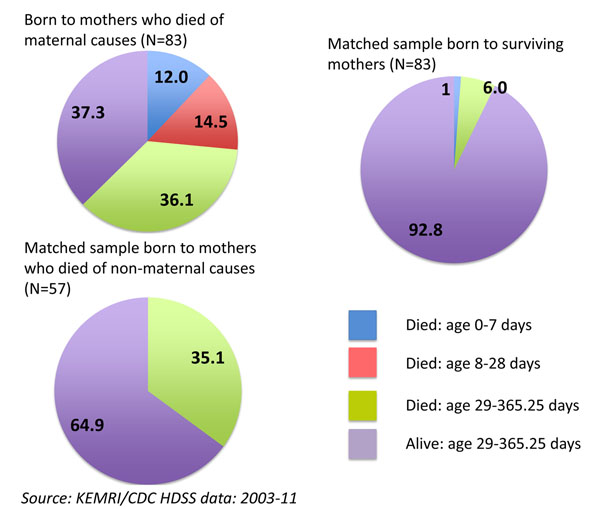
Survival status of live births till age 1 year by mother’s survival status

Between 2003-11, the HDSS recorded a total of 4749 deaths of women between the ages of 15-49 years. Of these, 391 were deaths due to maternal causes. Neonatal and infant survival varied significantly between these groups of women. About one-quarter of live births (26.5%) to women who died of maternal causes died before reaching one month of age (that is, between 0-28 days), compared to no deaths before age one month among live births to matched women who died of non-maternal causes, and one death among live births to matched surviving women (Figure 6). By age 1 year, only one-third of live births to women who died of maternal causes were still alive (37.3%), compared to about two thirds of live births to women who died of non-maternal causes before the child reached age 1 year (64.9%), and almost all live births to surviving women (92.8%).

#### Children’s care and feeding

Surviving older children also suffered. Three-quarters of household members who switched where they ate before and after the maternal death were children under the age of 18. In many cases children shifted to their grandmothers’ home or were fostered out to other relatives. This fostering caused trauma not only for the children, but also for the other adults in the home. The care of young infants following the maternal death also had ramifications for older children in the household who sometimes got less care. In one household, for example, the maternal grandmother noted that the extra care required by the orphaned infant and other young children now in her care meant that she had less time to care for her own two youngest sons, who were still living on the homestead and going to school. Older children also had to increasingly help with household tasks, leaving them less time to do their homework. Another respondent noted:

*“Now the tasks can’t be done as they used to be, for instance the children used to come from school and [find] things already done,…now...Because the grandmother is one person, it will force the children to join hands to have the duties done”* (Husband of the deceased).

#### Children’s schooling

About half the population of the surveyed households was of school-going age, which, in this area, is from 5-24 years of age, taking into account tertiary education as well as the fact that children often start and end school at late ages. The vast majority (85.7%) was in school when their mother was alive. While we do not have survey data on children’s schooling after a maternal death, the qualitative data offer evidence that schooling was disrupted by the death.

Several respondents noted that children typically missed school after their mother died because the household could no longer afford to pay school fees due to the loss of the deceased mother’s income, and because reduced household income went toward hiring casual labor. As one father noted:

“…Truly speaking, during the time you bring in a casual laborer, and he wants a thousand and over or two thousand shillings at the same time a child is sent home for exam fees PTA [Parent Teacher Association] money things like that and the money you have, had been given to the hired person, it will force the child to stay at home for sometime while you are still looking for the money. And if she [the mother] was there, you could not have hired anybody but instead the money could have gone to school…”

The added responsibilities older children shouldered once the mother died exacerbated this situation. Several respondents were anxious about how these responsibilities and gaps in schooling would negatively impact their children’s overall education. As noted by a brother-in-law of the deceased and echoed by others, *“…all the roads were blocked…*” and they felt *“…at a standstill…*” because they were unable to continue their children’s education as before.

### Emotional loss and coping mechanisms

#### Emotional loss

Parents’ emotional loss often expressed itself as anger and pain, such that they would *“…reach a point where the anger overcomes you…when you have certain thoughts*” (Mother of deceased). Parents of the deceased broke down in tears several times during the discussions, talking about how “*…bad feelings come whenever I do these tasks because she should be doing this. Even when sweeping I think that if she was there she could be the one sweeping and I preparing tea*” (Mother of deceased). Another parent referred to the year his daughter died as “*the year of suffering two thousand and eleven*” (Father of deceased).

Husbands expressed the loss of daily companionship:

“…you’ve lost a companion, because that person you are with at home, when you wake up, then you have to ask her “ichiew nadi” [a morning greeting similar to ‘good morning, how was your night’]? …And also she has to greet you, when you are going out she would ask, “now that you leaving where are you going” and when you are back she will recognize your presence…Therefore it must be that all your thoughts are with her all the time, at the time she leaves you and is not there they also go with your thoughts. They go with your thoughts until sometimes if someone speaks to you about them in a certain way, you just feel like, you feel that pain come back, that so and so has already left us.”

Discussion participants also described the emotional ramifications for children. As one respondent explained to the discussion moderator:

*“But now that joy they [the children] had has reduced. Now, it is a must that you tell the child that “when you come back, you will go to fetch water, when you come back, there is no firewood”. These are things that are done without the joy they had in the past, they now just see their mother’s [the deceased] part [that is, they are aware they are doing work their mother used to do].”* (Brother-in-law of deceased).

#### Coping and support mechanisms

The church, family members, friends, and neighbors provided material goods, food, labor and financial support for the deceased’s funeral and during the mourning period. The survey data show that 87% of the households that suffered a maternal death received some form of financial support for the funeral from family members, and two-thirds were able to fundraise in the community. Yet, a quarter of households were still compelled to sell assets, and about 15% resorted to moneylenders^b^.

Unfortunately, most of the support – financial and emotional – waned after the mourning period as family and community went back to their daily routines. As one respondent noted: *“People have to assist you with the burden of bringing the deceased home and the burial that ends there*, *but what can assist you later is not there.”* (Mother-in-law of deceased). Another agreed: “*But when death occurs a person brings fifty shillings*, *the other one brings a hundred shillings and so on and so forth*, *somebody comes with sugar the other person the same way. Things happen when death occurs. But after burial that is the end*, *it is now you.”* (Mother-in-law of deceased).

Surviving household members thus had to cope largely unaided with the post-mourning aftermath of reorganizing their households and providing for their children. As one respondent noted, laughing: “*You calm yourself because there is nothing else you can do*” (Brother-in-law of deceased). Another noted: *“ ‘sani koro eka ayawo bada’[a Luo saying that means one has intensified one’s effort].”* (Husband of deceased). Surviving household members continued the best they could. Many turned to religion, noting that they could not question or refuse “*…what God has given…*” (Husband of deceased), and that they had to continue their lives, albeit *“…with a heavy heart”* (Mother of deceased).

## Conclusion

We examined the consequences of maternal death in Siaya County in western Kenya, using a combination of surveillance, survey and qualitative data. Our findings documented the profound consequences of a maternal death for children and households. Consistent with other studies [9], we found that the risks of death to neonates and infants are significantly higher for children born to a mother who dies of maternal causes compared to those born to surviving mothers or to mothers who die within the first year after birth of non-maternal causes.

Maternal death created a ‘disruption chain’ in the household. Increased demands of household work and childcare meant that other adults spent less – and interrupted – time on economic activities; they also were compelled to hire helpers for farming. Mothers-in-law were disproportionately affected, shouldering the major share of the burden of feeding additional family members, as well as a large part of the responsibility of household, childcare and farming tasks after a maternal death. The disruptions caused by these changes had consequences for children’s emotional health, schooling, and care, echoing findings from other studies about the consequences for young children and their households of the death of a mother from other causes [17], including HIV/AIDS [18]. Our qualitative data illustrated also the distress experienced by bereaved husbands, orphaned children, parents and parents-in-law. Finally, there was little help and support to be had after the funeral and mourning period were over.

This study had certain limitations. First, the study is based on a small sample from one area in Kenya. As such it may not be generalizable. Still, other research suggests that households in the rest of Kenya, as well as in other developing countries with high maternal mortality, likely face similarly devastating consequences when a young woman dies in childbirth [10,19,20]. Second, since the reporting of household functioning pre-maternal death is retrospective, theoretically it could be idealized post-death. Unfortunately we did not collect similar data from comparison households to test for this possibility. Still, because of the relatively small time gap between the death and the interview, we expect such idealization to be minimal.

Despite these limitations, our study illustrates vividly that maternal death has detrimental consequences that ripple out from the immediate woman’s spouse and children to the entire household, and across generations. Though maternal death may not be the largest cause of death, still, the shock of a maternal death because of its unexpected nature may exacerbate the struggles faced by the surviving family, particularly if they also have to care for a newborn. Other work in sub-Saharan Africa on the effects of severe obstetric morbidity shows also that even if women don’t die immediately and unexpectedly at or soon after childbirth, those who suffer severe obstetric morbidity are more likely to die thereafter [21,22]– and their infants more likely to die [21] – than women who do not suffer such morbidity during pregnancy. Finally, even if women who suffer severe obstetric morbidity do not die, their suffering can have severe detrimental consequences for household functioning and child welfare [23-25].

Our study joins this work on severe obstetric morbidity in emphasizing the need to address the poor use and low quality of maternal and neonatal care in Kenya and elsewhere [26]. The government and other organizations in Kenya already have experience with several models of providing care to infants, older children and households made vulnerable because of parental deaths from other causes such as AIDS [27-30]. Our study suggests that infants and young children orphaned due to maternal death, and their familial caretakers, would benefit from being explicitly included in these and similar efforts.

## Competing interests

The authors declare that they have no competing interests.

## Authors’ contributions

RPP led the conception and design of the study, led the design of the qualitative and quantitative instruments, conducted part of the qualitative and quantitative analysis, wrote the manuscript, and approved the final draft. SO contributed to the design of the study, led and supervised the quantitative and qualitative fieldwork, conducted the quantitative analysis in Stata and SAS, contributed to the writing of the manuscript, and contributed to the qualitative analysis in NVivo. RK contributed to the study design, conducted the qualitative analysis in NVivo, and contributed to drafting the manuscript. RR contributed to the design of the qualitative instruments, conducted part of the qualitative analysis, and contributed to drafting the manuscript. AK contributed to the study design, was involved in drafting the manuscript, and contributed to the quantitative analysis in Stata. FOO contributed to the conception and design of the study and its implementation. KL contributed to the conception and design of the study and its implementation. KS contributed to the conception and design of the study and to drafting and modifying the manuscript.

## Authors’ information

RPP is a demographer with a doctoral degree from the Johns Hopkins Bloomberg School of Public Health and over 20 years experience in research and programs on gender inequality and reproductive health; RPP was a lead researcher on this paper. SO is a research scientist based at KEMRI/CDC who supervised the fieldwork and was intimately engaged in conducting the study, reading manuscripts, and conducting quantitative and qualitative analyses. RK was a research scientist at Family Care International’s Kenya office at the time of the study, and worked closely with SO in implementing the study, interpreting the results, and conducting the qualitative data analysis. RR is currently a doctoral student at the Johns Hopkins Bloomberg School of Public Health and has many years experience conducting qualitative research on reproductive health; she was instrumental in creating the discussion guide and trained field workers in Kenya on using the guide. AK is a health economist at ICRW, with expertise in quantitative analysis, and trained fieldworkers in implementing the quantitative instrument for this study. FOO heads the KEMRI/CDC HDSS in Kisumu, and KL headed the KEMRI/CDC Research and Public Health Collaboration in Kisumu at the time of this study, and both were engaged in the study’s conception, design and implementation. KS is a senior program officer in FCI, based in New York, and with many years of experience in managing programs on maternal health in Kenya.

## Endnotes

^a^Data on births can be used to capture additional, potential maternal deaths that may have been missed by the verbal autopsy method, specifically, by identifying women who gave birth and died within 42 days of the birth. ^b^Each household could have received support for funeral expenses from multiple sources, thus the percentages are more than 100%. A different, forthcoming paper, discusses these expenses in more detail. ^c^We found no published data on causes of death in our area covering the study period. KEMRI/CDC’s HDSS estimates that between 2003-11, maternal death accounted for just under 10% of all deaths to women 15-49 years of age.

## Supplementary Material

Additional file 1 Click here for file

## Declarations

This article has been published as part of *Reproductive Health* Volume 12 Supplement 1, 2015: True costs of maternal death. The full contents of the supplement are available online at http://www.reproductive-health-journal.com/supplements/12/S1. Publication charges for this supplement were funded by Family Care International and the FXB Center for Health and Human Rights.
