# Supplementary material for: Continuing with “…a heavy heart” - consequences of maternal death in rural Kenya
Source: Reprod Health. 2015 May 6;12(Suppl 1):S2. doi: 10.1186/1742-4755-12-S1-S2 (PMC4423749; doi:10.1186/1742-4755-12-S1-S2)
Supplement: Additional file 1 [file 1742-4755-12-S1-S2-S1.pdf]

Referee's comments to the authors– this sheet WILL be seen by the author(s) and published with the article

Title	Continuing with a "Heavy Heart" - consequences of maternal death in rural Kenya: a multi-method study
Author(s)	Rohini Prabha Pande, Sheila Ogwang, Robinson Karuga, Radha Rajan, Aslihan Kes, Frank O. Odhiambo, Kayla Laserson, and Kathleen Schaffer
Referee's name	Cynthia Stanton

When assessing the work, please consider the following points, where applicable:

1. Is the question posed by the authors new and well defined?
2. Are the methods appropriate and well described, and are sufficient details provided to replicate the work?
3. Are the data sound and well controlled?
4. Does the manuscript adhere to the relevant standards for reporting and data deposition?
5. Are the discussion and conclusions well balanced and adequately supported by the data?
6. Do the title and abstract accurately convey what has been found?
7. Is the writing acceptable?

Please make your report as constructive and detailed as possible in your comments so that authors have the opportunity to overcome any serious deficiencies that you find and please also divide your comments into the following categories:

- Major Compulsory Revisions (which the author must respond to before a decision on publication can be reached)
- Minor Essential Revisions (such as missing labels on figures, or the wrong use of a term, which the author can be trusted to correct)
- Discretionary Revisions (which are recommendations for improvement but which the author can choose to ignore)

Where possible please supply references to substantiate your comments.

When referring to the manuscript please provide specific page and paragraph citations where appropriate.

General comments: This paper takes on the assessment of disruptions in household behavior and infant survival following maternal death in rural Kenya. The authors have used existing data from a demographic surveillance site, their own household survey, all complemented by qualitative interviews with surviving household members. They are to be congratulated for their comprehensive design. Below are my substantive and editorial comments and suggestions.

Major compulsory revisions:
1) I suggest that the authors might want to consider including in their review of the literature the work of V. Filippi, K. Storeng and colleagues regarding the consequences and follow up of Maternal Near Miss cases in West Africa. Although this series of studies does not refer to maternal deaths, it strengthens the story in Kenya by showing that even if the woman survives a major obstetric complication, women and households have many negative consequences to address.
2) My biggest problem in reading this paper is interpreting to what the results are compared. For example, infant survival among maternal deaths is described relative to births to non-maternal deaths and to births with surviving mothers, which makes for a powerful comparison. Results re: child care, child schooling, emotional loss, support mechanisms and disruptions are strictly descriptive and restricted to households having experienced a maternal death. I suggest the authors state their specific research objectives more clearly in the background section of the paper – including what the comparison groups are for each research question.

(continue on the next sheet)

Continued:
3) Regarding the descriptive results related to disruption in the household, my impression is that such disruption comes as a surprise to the authors and is negative; one gets the sense that ideally there should not be any disruption. Isn't it a given that there will be disruptions when an adult dies in a household with children of any age? How are the consequences of a maternal death different than the consequences of an HIV-related (which is more common in Kenya) or other non-maternal death? To me, the gist of your paper is that there is something uniquely bad about maternal deaths but you do not have the data to support that statement except for the issue of infant survival. Since I assume you do not have disruption data from households with non-maternal deaths or surviving mothers, it would be important to at least tell readers what percent of all adult female deaths in your study area are due to maternal causes. If maternal death is the leading cause of adult female death, then you have a stronger case to make that household disruption and other consequences are an important public health issue meriting interventions that are specific to maternal death. Other than provision of food to children (as is done in households with HIV positive children), I do not know what types of intervention the authors have in mind. A type of social security payment?

Editorial comments:
Page 4 and the first paragraph of page 5: The results from the literature from Nepal, Tanzania, Haiti and Kenya do not state to whom the increased risks are relative. For example, in Haiti: 55% higher odds of experiencing a death…. (relative to what other group? Non-maternal deaths? Surviving mothers?)

Page 6, 3rd paragraph: Society is polygamous and men may have more than one wife – is redundant.
Page 11, first paragraph, 2nd sentence: cohabiting is mentioned twice in this sentence.
Table 1: please explain why the number of people in interviewed households is important – is this the number of people your interviewers talked to to obtain the info in Table 1?
Page 11, Backround characteristics: include in the text the number of maternal deaths
Figure 7 and Page 16, 2nd paragraph: Information is provided from the demographic surveillance site but there are no n's in either the text or the graphic. The reader needs at least n's of births, n's of adult female deaths and n's of maternal deaths.

Referee's comments to the authors– this sheet WILL be seen by the author(s) and published with the article

Title	Continuing with a "Heavy Heart" - consequences of maternal death in rural Kenya: a multi-method study
Author(s)	Rohini Prabha Pande, Sheila Ogwang, Robinson Karuga, Radha Rajan, Aslihan Kes, Frank O. Odhiambo, Kayla Laserson, and Kathleen Schaffer
Referee's name	Jennifer Blum

When assessing the work, please consider the following points, where applicable:

1. Is the question posed by the authors new and well defined?
2. Are the methods appropriate and well described, and are sufficient details provided to replicate the work?
3. Are the data sound and well controlled?
4. Does the manuscript adhere to the relevant standards for reporting and data deposition?
5. Are the discussion and conclusions well balanced and adequately supported by the data?
6. Do the title and abstract accurately convey what has been found?
7. Is the writing acceptable?

Please make your report as constructive and detailed as possible in your comments so that authors have the opportunity to overcome any serious deficiencies that you find and please also divide your comments into the following categories:

- Major Compulsory Revisions (which the author must respond to before a decision on publication can be reached)
- Minor Essential Revisions (such as missing labels on figures, or the wrong use of a term, which the author can be trusted to correct)
- Discretionary Revisions (which are recommendations for improvement but which the author can choose to ignore)

Where possible please supply references to substantiate your comments.

When referring to the manuscript please provide specific page and paragraph citations where appropriate.

General comments:
I found this paper to be very interesting, but it is really long. At times, I felt that it was a bit repetitive and perhaps it can be shortened by looking closely for repetitions in the various sections. I understand that qualitative studies often generate long papers, but I think they may lose readers if they don't tighten it up a bit. The documents I received to review did not include the figures, so I was unable to review them. But, again, I wonder if 7 figures is necessary. I would like to see more from the children's perspectives, if other data are available. Although, I mention this with the caveat that I also feel that the paper is long so adding anything additional may not contribute to shortening the document.

I think the analysis is methologicially strong and well described.

Supplement Editor's comments

In addition to the reviewers´ recommendations, I wonder if you can rewrite the Conclusions of the abstract. I consider that you are giving recommendations that are some beyond the results of your study. I am suggesting you to give more straightforward conclusions that can be supported by the results of your study.
